# Modulation by Ethanol of Cigarette Smoke Clastogenicity in Cells of Adult Mice and of Transplacentally Exposed Fetuses

**DOI:** 10.1371/journal.pone.0167239

**Published:** 2016-12-01

**Authors:** Roumen Balansky, Sebastiano La Maestra, Rosanna T. Micale, Marietta Iltcheva, Krassimir Kirov, Silvio De Flora

**Affiliations:** 1 National Center of Oncology, Sofia, Bulgaria; 2 Department of Health Sciences, University of Genoa, Genoa, Italy; University of Kentucky, UNITED STATES

## Abstract

Cigarette smoke (CS) and ethanol (EtOH) are known to synergize in the causation of cancers of the upper aerodigestive tract and of the liver. Little is known about possible interactions between these agents in other organs. These premises prompted us to evaluate the clastogenic effects resulting from the inhalation for 3 weeks of mainstream CS and oral administration of EtOH, which were tested either individually or in combination in cells of adult BDF_1_ mice and their fetuses. CS exerted clastogenic effects in haematopoietic cells of adult male mice by increasing the frequency of micronucleated erythroid cells both in bone marrow and in peripheral blood as well as the frequency of micronucleated and polynucleated pulmonary alveolar macrophages. Likewise, exposure to CS of pregnant mice resulted in a clastogenic damage in maternal bone marrow cells and in the liver and peripheral blood of their fetuses. Under all experimental conditions, EtOH was consistently devoid of clastogenic effects when given alone. In adult mice, EtOH exhibited a mild stimulating effect on the clastogenicity of CS in haematopoietic cells, while an opposite effect was observed in the respiratory tract, where EtOH attenuated the cytogenetic alterations induced by CS in pulmonary alveolar macrophages. At variance with the mild synergism observed in haematopoietic cells of adult mice, EtOH inhibited the clastogenicity of CS in the liver and peripheral blood cells of transplacentally exposed fetuses. Therefore, the effects of EtOH in CS-exposed mice show different trends depending both on the life stage and on the cells analyzed.

## Introduction

Individually, cigarette smoke (CS) and ethanol (EtOH) are two major risk factors for cancer and for a variety of other chronic degenerative diseases. CS is positive in virtually any short-term test predictive of carcinogenic effects in which this complex mixture has been assayed [1–5]. Smoking in humans has been associated with cancers targeting a number of tissues in the respiratory system, including the nasal cavity and paranasal sinuses, pharynx (nasopharynx, oropharynx and hypopharynx), larynx, and above all trachea and lung, as well as in the urinary tract (kidney pelvis, ureter, and bladder), digestive system (oral cavity, oesophagus, stomach, colon-rectum, liver, and pancreas), reproductive tract (ovary and uterine cervix), and hematopoietic system (myeloid leukemia) [4]. CS carcinogenicity is due to composite mechanisms affecting all stages of the carcinogenesis process, also depending on the fact that combustion of tobacco leaves generates large amounts of free radicals and more than 5000 identified chemical compounds belonging virtually to any chemical family, 73 of which have been evaluated by IARC to be carcinogenic in humans and/or experimental animals [6]. On the other hand, EtOH is a single chemical compound that exerts its toxicity via its metabolite acetaldehyde, which is generated by cytoplasmic alcohol dehydrogenase, and reactive oxygen species (ROS),which are predominantly generated by the microsomal cytochrome P450 2E1 (CYP2E1). In addition to ethanol oxidation, CYP2E1 plays a role in the metabolic activation of many carcinogens, including *N*-nitrosamines, benzene and aniline [4]. Although EtOH is generally regarded as non-genotoxic [7,8], exocyclic etheno-DNA adducts are formed in tissues of individuals consuming EtOH, primarily via induction of ROS-generating CYP2E1 [9], and acetaldehyde reacts with DNA to form a variety of different types of DNA adducts [10]. In addition, EtOH alters epigenetics by changing DNA and histone methylation and acetylation [11]. Like CS, EtOH is classified as a human carcinogen, but its targets are limited to the proximal aerodigestive tract (oral cavity, pharynx, larynx), to the digestive tract (oesophagus, liver, colorectum) and to female breast [8].

The combination of CS and EtOH deserves a special interest because it has been estimated that the 80 to 95% of heavy alcohol users are also smokers [12]. The synergism between CS and EtOH could be reproduced both *in vitro* by evaluating the mutagenicity of combined agents in strain YG1029 of *Salmonella typhimurium* [13] and *in vivo* by evaluating the cytogenetic damage in bone marrow cells of male BD6 rats [7] and the levels of bulky DNA adducts in the oesophagus of BD_6_ rats co-exposed to CS and EtOH [14]. However, a study in Chinese hamsters failed to point out any increase of structural chromosome aberrations or sister chromatid exchanges in bone marrow cells of animals treated, either individually or in combination, with CS and EtOH [15]. In humans, an elevated frequency of micronucleated (MN) buccal mucosa cells was observed in a group of smokers and alcohol drinkers but not in subjects exposed to either agent individually [16]. In addition, it is well documented that CS and EtOH synergize in the causation of cancers of the oesophagus and in general of the upper aerodigestive tract. Indeed, heavy drinking and smoking are considered to be the most important risk factors for head and neck cancer [17]. There are some indications also regarding the synergism of these agents in causing liver cancer [18].

We developed a new experimental system in which mainstream CS (MCS) becomes convincingly carcinogenic to strain H mice when exposure covers the first 4 months of life, starting at birth, followed by 3–4 months of recovery in filtered air [19]. This system was extensively applied to evaluate both safety and efficacy of a number of dietary and pharmacological agents in MCS-related carcinogenesis [20]. Using the same model, we recently evaluated the ability of EtOH to modulate MCS carcinogenicity. Especially in mice exposed both transplacentally and in the postnatal life, EtOH administration was associated not only with liver damage but also with pro-angiogenetic effects in the lung by stimulating the proliferation of blood vessels at 8 months of life. In addition, these mice developed pulmonary emphysema, alveolar epithelial hyperplasias, microadenomas, and tumors. On the other hand, EtOH interfered in the lung carcinogenesis process resulting from the concomitant exposure of mice to MCS by significantly attenuating some MCS-related preneoplastic and neoplastic lesions in the respiratory tract, such as alveolar epithelial hyperplasia, microadenomas, and even malignant tumors. Moreover, at 4 months of life EtOH administration resulted in a significant mitigation of the systemic MCS clastogenicity [21].

These results prompted us to design an *ad hoc* study aimed at exploring in detail the effects of EtOH on MCS clastogenicity in the bone marrow, peripheral blood, and pulmonary alveolar macrophages (PAM) of male adult BDF_1_ mice treated, either individually or in combination, with EtOH and MCS at various doses and exposure times. In addition, we exposed female mice of the same strain to EtOH and/or MCS throughout pregnancy and evaluated the clastogenic damage both in maternal bone marrow erythrocytes and in liver and peripheral blood erythrocytes of their offsprings. The results obtained provide evidence for a complex interaction between oral EtOH and inhaled MCS. In fact, the main outcome was an antagonistic effect of EtOH towards MCS clastogenicity in the respiratory tract of adult mice and in hematopoietic cells of their fetuses, contrasting with an enhancement of MCS clastogenicity in bone marrow and peripheral blood of adult mice.

## Materials and Methods

### Ethics statement

Housing, breeding, and treatment of mice were in accordance with the European Community Directive No. 2010/63/UE. The study was approved by the ethics committee of the National Center of Oncology (Sofia, Bulgaria). All efforts were made to ameliorate animal suffering. Animal sacrifice was performed by CO2 asphyxiation followed by cervical dislocation.

### Mice

BDF_1_ (C57BL x DBA_2_) mice were bred and maintained in the animal house of the National Center of Oncology (Sofia, Bulgaria). The mice were housed in Makrolon^™^ cages on sawdust bedding and maintained on standard rodent chow (Kostinbrod, Sofia, Bulgaria) and drinking water *ad libitum*. The animal room temperature was 23 ± 2°C and the relative humidity was 55%, with a 12 h day–night cycle.

### Administration of EtOH and exposure to MCS

EtOH (Sigma Chemical Co. St. Louis, MO, USA) was added to tap water at the concentration of either 5% or 10% (v/v) and given as the only source of drinking water to mice. A whole-body exposure to MCS for either 60 or 90 min/day was achieved as previously described [22] by burning Bulgartabac Sredetz cigarettes, having a declared content of 13 mg tar and 1.0 mg nicotine each, and delivering 16 mg CO. The average concentration of total particulate matter in the exposure chambers was 673 mg/m^3^ air.

### Study in adult male mice

A total of 70 BDF_1_ male mice, aged 2.5 months and weighing 27–29 g, were used for evaluating the individual and combined effects of EtOH and MCS on cytogenetic alterations in bone marrow polychromatic erythrocytes (PCE), peripheral blood normochromatic erythrocytes (NCE), and pulmonary alveolar macrophages (PAM). The following 7 groups of mice, each composed of 10 mice, were used: *Group A*. Sham-exposed mice, kept in filtered air; *Groups B* and *C*. Mice exposed to MCS for either 60 or 90 min/day; *Groups D* and *E*. Mice receiving either 5% or 10% EtOH with the drinking water; *Group F*. Mice exposed to MCS for 60 min/day and receiving 5% EtOH with the drinking water; *Group G*. Mice exposed to MCS for 90 min/day and receiving 10% EtOH with the drinking water. All treatments lasted 3 weeks.

The peripheral blood was collected from the tail lateral vein of all 70 mice after 1, 5, 10, 15, and 20 days of treatment. The blood samples were smeared onto slides (two slides/mouse) and stained with May–Grünwald–Giemsa according to Schmid [23]. The frequency of MN NCE was evaluated by analyzing microscopically 50,000 NCE in each mouse.

After 3 weeks of treatment, all 70 mice were killed by cervical dislocation. Bronchoalveolar lavage (BAL) was performed by lavaging the lungs with two 5 ml aliquots of cold 0.15 M NaCl infused via a cannula inserted into the trachea. BAL cells were washed twice with RPMI 1640, spun in a cytocentrifuge, fixed with methanol, and stained for 10 min with a 10% Giemsa solution [24]. The cytology of BAL cells was evaluated, and the frequency of MN and polynucleated (PN) PAM was evaluated by analyzing microscopically 2,000 PAM in each mouse. Immediately after, the left femur from each mouse was removed and dissected. Bone marrow cells were collected, smeared on duplicate slides, air dried, and stained with May–Grünwald–Giemsa. The PCE/NCE ratio was assessed by scoring 200 erythrocytes, and the frequency of MN PCE was evaluated by analyzing microscopically 4,000 PCE in each mouse.

### Study in pregnant mice and their fetuses

This study used pregnant BDF_1_ mice and their fetuses. Four groups of pregnant mice, each composed of 3 mice, were treated as follows: *Group H*. Sham-exposed mice, kept in filtered air; *Group I*. Mice exposed to MCS for 60 min/day; *Group J*. Mice receiving 5% EtOH with the drinking water; *Group K*. Mice exposed to MCS for 60 min/day and receiving 5% EtOH with the drinking water. All treatments started when the females were separated from the mating males and lasted until day 18 of gestation. At that time, the mice were killed by cervical dislocation. The left femur from each pregnant mouse was removed and dissected, and bone marrow cells were used to evaluate the PCE/NCE ratio and the frequency of MN PCE, as described above.

The litter of each pregnant mice was composed on an average of 9.3 fetuses, without any significant difference among the 4 experimental groups, for a total of 112 fetuses. The peripheral blood and liver were removed from each fetus, as previously described [25]. The frequency of MN PCE was evaluated both in the liver and peripheral blood of all fetuses, and the PCE/NCE ratio was assessed in each fetus liver.

### Statistical analysis

All results are expressed as means ± SE within each group of mice. Comparisons between groups regarding cytogenetic parameters were made by ANOVA followed by Student’s *t* test for unpaired data. *P* values lower than 0.05 were regarded as statistically significant.

## Results

### Cytogenetical damage in bone marrow of adult male mice

As shown in Table 1, administration for 3 weeks of either 5% or 10% EtOH did not significantly increase the frequency of MN PCE in bone marrow and did not change the PCE/NCE ratio. Exposure to MCS significantly increased the frequency of MN PCE, without altering the PCE/NCE ratio and without appreciable differences in mice exposed for either 60 min or 90 min/day. Administration of 5% EtOH to mice exposed to MCS for 60 min/day did not affect the MCS-related cytogenetic damage but it slightly increased the PCE/NCE ratio. Administration of 10% EtOH to mice exposed for 90 min/day significantly increased the frequency of MN PCE. Such a combination stimulated proliferation of erythroid cells, as shown by a significant increase of the PCE/NCE ratio.

**Table 1:** Cytogenetical damage in the bone marrow and in PAM of adult male BDF_1_ mice exposed to MCS and/or receiving 5% EtOH with the drinking water for 3 weeks.

Treatment	Bone marrow		BAL cells				
	MN PCE (‰)	PCE/NCE	PAM(%)	PMN(%)	MNC(%)	MN PAM (‰)	PN PAM (‰)
Controls (Sham)	2.8±0.22	1.0±0.06	94.2	5.0	0.8	0.2±0.11	15.1±0.75
5% EtOH	3.4±0.34	1.0±0.12	92.4	5.5	2.2	0.6±0.22	16.3±1.35
10% EtOH	3.2±0.39	1.3±0.14	78.6	14.4	7.0	0.4±0.19	20.6±5.39
MCS 60min/day	4.6±0.44^b^	1.0±0.08	50.7	46.1	3.1	2.2±0.62^c^	79.7±7.13^c^
MCS 90min/day	4.1±0.53^a^	1.0±0.07	51.9	44.3	3.9	1.1±0.32^b^	66.1±5.50^c^
MCS 60min/day +5% EtOH	4.1±0.54^a^	1.3±0.12^a^^,^^d^	55.2	41.1	3.7	0.5±0.35^d^	58.4±5.50^c^^,^^d^
MCS 90min/day +10% EtOH	5.5±0.36^c^^,^^d^	1.7±0.07^c^^,^^e^	55.1	41.2	3.7	0.2±0.12^d^	48.9±5.94^c^^,^^d^

The results are means ± SE within each group of 10 mice.

Abbreviations: MN, micronucleated; PN, polynucleated; PCE, polychromatic erythrocytes; NCE, normochromatic erythrocytes; BAL, bronchoalveolar lavage; PAM, pulmonary alveolar macrophages; PMN, polymorphonucleates; MNC, mononuclear cells.

Statistical analysis:

^a^ *P <* 0.05,

^b^ *P <* 0.01, and

^c^*P* < 0.001, as compared with Sham;

^d^ *P* < 0.05 and

^e^ *P* < 0.01, as compared with the corresponding MCS.

### Cytogenetical damage in peripheral blood of adult male mice

**Fig 1:**
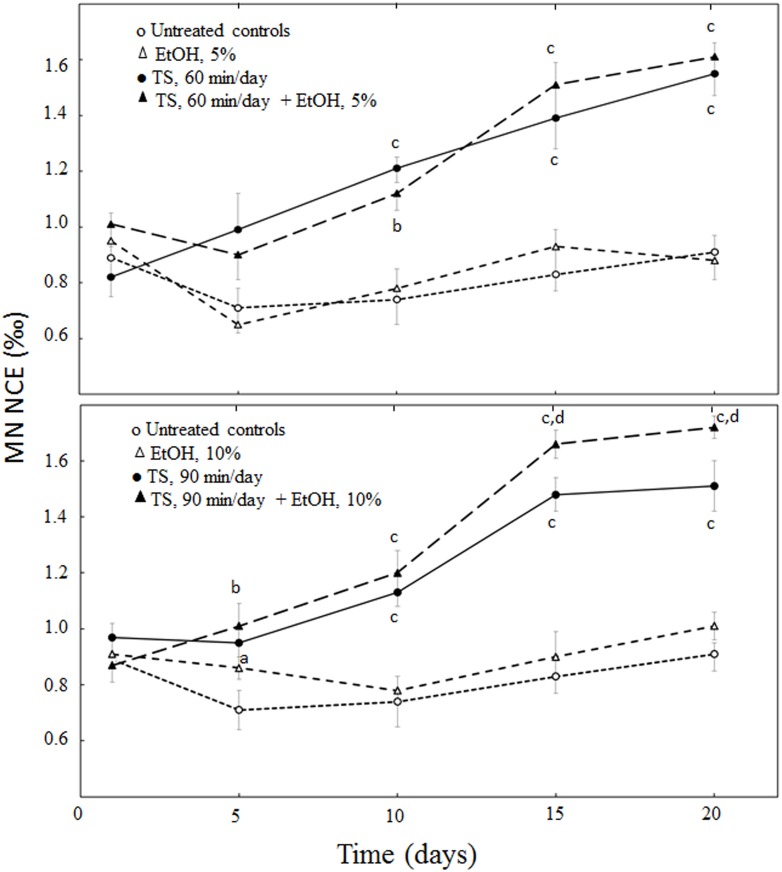
Frequencies of MN NCE in the peripheral blood of adult male mice. The mice were either untreated (controls) or exposed to MCS for 60 min/day and/or receiving 5% EtOH with the drinking water (upper panel) or either untreated (controls) or exposed to MCS for 90 min/day and/or receiving 10% EtOH with the drinking water (bottom panel). Statistical analysis: ^a^*P* < 0.05, ^b^*P* < 0.01, and ^c^*P* < 0.001, as compared with the corresponding controls;: ^d^*P* < 0.05, as compared with the corresponding MCS.

The results relative to the time-course monitoring of MN NCE frequency in the peripheral blood of adult male BDF_1_ mice are summarized in Fig 1, along with their statistical analysis. Exposure to MCS for either 60 min or 90 min/day resulted in a progressive and significant clastogenic damage, as compared with sham-exposed mice. Administration of either 5% or 10% EtOH to smoke-free mice did not change the frequency of MN NCE at any time point. Administration of 5% EtOH to mice exposed to MCS for 60 min/day did not significantly affect the MCS-related clastogenic damage. However, consistently with the effect observed in bone marrow after 3 weeks, 10% EtOH significantly enhanced clastogenicity after 15 and 20 days of treatment when it was combined with exposure to MCS for 90 min/day.

### Cytogenetical damage in BAL cells of adult male mice

Table 1 shows the cellularity of BAL cells and the frequency of MN and PN PAM after exposure of mice to MCS and/or administration of EtOH for 3 weeks. The BAL cytology was not appreciably altered by administration of 5% EtOH, whereas 10% EtOH increased the relative proportion of PMN and MNC at the expenses of PAM. Due to the increase of PMN, the relative proportion of PAM was considerably decreased in MCS-exposed mice, irrespective of administration of EtOH. Treatment with EtOH, either 5% or 10%, did not affect the frequency of MN and PN cells, as compared with sham-exposed mice. Exposure to MCS for 90 min/day and even more exposure for 60 min/day significantly increased the frequency of both MN and PN PAM. Such an effect was significantly attenuated by co-treatment of MCS-exposed mice with EtOH, to such an extent that the frequency of MN PAM in MSC-exposed mice receiving EtOH with the drinking water was not significantly different from that of sham-exposed mice.

### Cytogenetical analyses in pregnant mice and their fetuses

The body weights of fetuses from sham-exposed pregnant mice, MCS-exposed mice, EtOH-treated mice and mice treated with both MCS and EtOH (mean ± SE) were 1.38 ± 0.03, 1.23 ± 0.04 (*P* < 0.01 compared to sham), 1.32 ± 0.03 (not significant), and 1.27 ± 0.04 (*P* < 0.05), respectively. Table 2 shows the results of cytogenetical analyses in bone marrow erythrocytes from pregnant mice and in the liver and peripheral blood erythrocytes from their fetuses. Presumably due to the fact that only 3 pregnant mice per group were available, the evident increase in the frequency of MN PCE in the bone marrow of MCS-exposed pregnant mice was not statistically significant, while the PCE/NCE ratio was significantly increased in this group. Administration of 5% EtOH to MCS-free mice was devoid of significant effects on the above parameters, while administration of 5% EtOH to MCS-exposed pregnant mice resulted in a significant and considerable (almost 3-fold) increase in the frequency of MN PCE as compared to sham-exposed mice.

**Table 2:** Cytogenetical damage in the bone marrow of BDF_1_ dams, exposed to MCS for 60 min/day and/or receiving 5% EtOH with the drinking water throughout pregnancy, and in the liver and peripheral blood of their fetuses.

	Pregnant mice		Fetuses		
	Bone marrow		Liver		Peripheral blood
Treatment	MN PCE (‰)	PCE/NCE	MN PCE (‰)	PCE/NCE	MN PCE (‰)
Controls (Sham)	1.3±0.75	1.4±0.37	2.7±0.31	0.8±0.10	2.5±0.47
MCS 60min/day	2.5±0.50	2.5±0.11^a^	5.5±0.60^b^	1.2±0.09^a^	7.9±0.57^b^
5% EtOH	1.4±0.63	1.6±0.46	2.3±0.26	0.9±0.06	2.2±0.28
MCS 60min/day +5% EtOH	3.3±0.59^a^	2.0±0.55	2.7±0.29^c^	1.3±0.08^b^	3.8±0.25^a^^,^^c^

The results are means ± SE within each group of mice.

Statistical analysis:

^a^ *P <* 0.01 and

^b^ P *<* 0.001, as compared with Sham;

^c^ *P* < 0.001, as compared with MCS.

The transplacental exposure of fetuses to MCS caused a significant increase of the PCE/NCE ratio as well as a clastogenic effect documented by an increased frequency of MN PCE in liver. Maternal EtOH did not cause any significant effect, and its administration to MCS-exposed pregnant mice inhibited the MCS-related increase in the frequency of MN PCE in fetus liver, without affecting the elevation of the PCE/NCE ratio. Similarly, a significant increase in the frequency of MN PCE was detected in the peripheral blood of fetuses whose dams had been exposed to MCS throughout pregnancy. Again, co-treatment of pregnant mice with EtOH significantly attenuated the MCS-induced clastogenic damage.

## Discussion

The results of the present study confirm the ability of MCS to induce clastogenic damage both in adult mice and in transplacentally exposed fetuses. Moreover, for the first time, they provide evidence for the lack of clastogenicity of EtOH in bone marrow cells and PAM of adult mice and in haematopoietic cells of fetuses as well. In addition, the data herein presented highlight the complexity of the interactions between MCS and EtOH, which depend on the life stage, the organ studied, and target cells.

In particular, exposure to MCS of adult male mice for 3 weeks resulted in a time-related increase of MN NCE in peripheral blood. Moreover, at the end of that period, increases in the frequencies of both MN PCE in bone marrow and of MN and PN PAM in the lower respiratory tract were observed in MCS-exposed mice. These effects reflect the capacity of MCS to induce chromosome breaks and malfunction of the spindle apparatus leading to formation of MN as well as disturbances in PAM homeostasis and division thereby resulting in the formation of PN cells. In separate experiments, exposure of mice for 60 min/day to the same MCS dose used in the present study resulted in a blood serum cotinine concentration, measured by ELISA, of 51.9 ± 1.8 ng/ml [S. La Maestra, unpublished data]. Likewise, exposure of female mice throughout pregnancy increased the PCE/NCE ratio in bone marrow. These results are in line with the findings of pioneer studies showing the increase of both MN PCE in the bone marrow [1] and MN NCE in the peripheral blood [2] of mice exposed whole-body to MCS. As previously documented [26], the induction and persistence of cytogenetic alterations in MCS-exposed mice display differential patterns depending on pharmacokinetic and metabolic mechanisms and especially on the lifespan of the targeted cells. In fact, after exposure of mice for 3 weeks, the frequency of MN PCE increases early but declines to background levels upon discontinuation of exposure to MCS due to the fast turnover of these highly proliferating cells. Appearance of MN in circulating NCE is slightly delayed and less intense but persists for an additional 3 weeks [26], consistently with the notion that the NCE half-life in the mouse peripheral circulation is about one month [27]. In contrast, MCS-induced cytogenetic alterations in PAM persist for at least 14 weeks [26], due the fact that these cells are extremely long-lived [28]. Likewise, the observed clastogenic damage in the offspring from MCS-exposed pregnant mice provides further evidence that the transplacental passage of genotoxic smoke components leads to the formation of MN PCE in the liver, which are thereafter released into the general circulation [25].

On the other hand, administration of either 5% or 10% EtOH for 3 weeks did not significantly increase the frequency of MN PCE in bone marrow and did not change the PCE/NCE ratio either in adult male mice or in pregnant female mice. Similarly, no increase of MN and PN PAM was detected in the respiratory tract, although the higher EtOH dose (10%) elicited some inflammatory response, as shown by an altered BAL cytology. The lack of clastogenicity of EtOH in the mouse model used was confirmed by the finding that maternal EtOH did not cause any significant effect in fetus cells. Note that exposure of rats to 5% EtOH resulted in blood EtOH concentrations similar to those seen in chronic alcoholics [29].

The possible modulation by EtOH of MCS-induced clastogenic damage was the major goal of the present study. Distinctive effects were observed in different cells of adult mice and in fetuses. In fact, EtOH further increased stimulation by MCS of MN PCE in the bone marrow of adult male mice as well as the MCS-induced increase of MN NCE in peripheral blood, but only at the higher EtOH dose tested and for longer daily exposures to MCS. Under these conditions, the PCE/NCE ratio was increased in bone marrow. Likewise, EtOH further increased the frequency of MN PCE and increased the PCE/NCE ratio in the bone marrow of pregnant mice exposed to MCS. These results suggest that, at high doses, EtOH has a mild enhancing effect on the systemic MCS clastogenicity in bone marrow. In contrast, irrespective of the EtOH dose and daily exposure time to MCS, EtOH considerably inhibited both the MCS-induced increase of PN PAM and even more dramatically that of MN PAM in the terminal airways of adult mice. Therefore, on the whole, the oral administration of EtOH increased the clastogenicity of MCS in both bone marrow and peripheral blood of adult mice, while an opposite effect was observed in the respiratory tract of the same mice.

These results are in agreement with the conclusions of studies in adult BD_6_ rats [7] and strain H mice [21]. It should be taken into account that epigenetic mechanisms provide an important contribution to the carcinogenicity of both CS and even more of EtOH. Furthermore, tumors probably originate from stem cells, whereas genotoxic alterations may affect other pulmonary cell types as well [30]. Nevertheless, it is noteworthy that modulation of clastogenic damage, as shown in the present study, correlates with modulation of lung tumors and other pulmonary histopathological alterations by EtOH in MCS-exposed mice [21]. Consistently with the above conclusion, a recent study in male albino Wistar rats showed that subcutaneous injections of nicotine dissolved in 25% EtOH are able to mitigate the damage to the lung, as compared to rats receiving nicotine in physiological saline. In fact, pulmonary histopathological alterations, such as emphysema, congestion and hemorrhage, were less marked in rats treated with both nicotine and EtOH compared to rats treated with nicotine only. In addition, occurrence of MCS-related oxidative damage to the lung was documented by increased levels of antioxidant enzyme activities (superoxide dismutase and catalase), which were less pronounced when nicotine was combined with EtOH [31]. Likewise, the highest levels of GSH were detected in the lungs of alcohol-addicted Wistar rats that were simultaneously exposed to CS and EtOH [32]. Interestingly, a metabolic cross-tolerance between nicotine and EtOH has been described in rats, suggesting that nicotine use may increase the elimination of EtOH, and that EtOH use may increase the elimination of nicotine [33]. In agreement with the above hypothesis, CS decreased acetaldehyde concentration after EtOH administration, while EtOH increased the rate of elimination of cotinine [34]. In mice, EtOH inhibited the pulmonary tumorigenicity of urethane [35], and disulfiram inhibited the clastogenicity of this CS constituent, which like EtOH is metabolized via the CYP2E1 pathway [36]. These findings support the hypothesis that EtOH may alter the metabolism of clastogenic MCS constituents in the lower respiratory tract.

Modulation of MCS clastogenicity by EtOH in mouse fetal liver was different from that observed in the haematopoietic cells of adult mice. In fact, EtOH considerably attenuated the MCS-induced clastogenicity in the liver, which in the prenatal life is a haematopoietic organ, a chemoprotective effect that could be also traced in the peripheral blood of fetuses exposed transplacentally. This finding may be ascribed to the distinctive EtOH metabolism in adults and fetuses. In fact, it has been shown that fetuses exposed to a 5% ethanol diet throughout gestation exhibit transplacental induction of a hepatic CYP2E1 that may possess different catalytic properties from the analogous adult enzyme [37]. When evaluating the effects of EtOH on fetal DNA, it should be also noted that EtOH administration to pregnant mice alters epigenetics by changing DNA and histone methylation and acetylation, which might affect the regulation of gene expression even following transplacental exposure [38].

In conclusion, inhaled MCS was confirmed to exert clastogenic effects in haematopoietic and respiratory cells of adult mice and in haematopoietic cells of mouse fetuses. Under all experimental conditions, EtOH was consistently negative when administered alone *per os*. However, EtOH treatment in combination with exposure to MCS modulated the clastogenicity of this complex mixture, with different trends depending both on the life stage, organ analyzed, and target cells. Namely, in adult mice EtOH exhibited a mild stimulating effect on the systemic clastogenicity of MCS, as evaluated by assessing the frequency of both MN PCE in bone marrow and MN NCE in peripheral blood. In contrast, EtOH attenuated the cytogenetic alterations induced by MCS in PAM of the same mice. This finding mechanistically supports the fact that, in contrast with the outcome of the interaction between MCS and EtOH in cells of the upper aerodigestive tract [14, 16–18], EtOH is able to attenuate MCS carcinogenicity in mouse lung [21]. Furthermore, EtOH inhibited the clastogenicity of MCS in the liver and peripheral blood haematopoietic cells of transplacentally exposed fetuses, presumably due to distinctive metabolic pathways involved in EtOH metabolism in the fetal life and in adults.
